# DNA Sequence Analyses Reveal Abundant Diversity, Endemism and Evidence for Asian Origin of the Porcini Mushrooms

**DOI:** 10.1371/journal.pone.0037567

**Published:** 2012-05-18

**Authors:** Bang Feng, Jianping Xu, Gang Wu, Md. Iqbal Hosen, Nian-Kai Zeng, Yan-Chun Li, Bau Tolgor, Gerhard W. Kost, Zhu L. Yang

**Affiliations:** 1 Key Laboratory of Biodiversity and Biogeography, Kunming Institute of Botany, Chinese Academy of Sciences, Kunming, Yunnan, People's Republic of China; 2 Department of Biology, McMaster University, Hamilton, Ontario, Canada; 3 Institute of Mycology, Jilin Agricultural University, Changchun, Jilin, People's Republic of China; 4 Systematic Botany & Mycology, Philipps-University Marburg, Marburg, Germany; 5 Graduate University of Chinese Academy of Sciences, Beijing, People's Republic of China; CNR, Italy

## Abstract

The wild gourmet mushroom *Boletus edulis* and its close allies are of significant ecological and economic importance. They are found throughout the Northern Hemisphere, but despite their ubiquity there are still many unresolved issues with regard to the taxonomy, systematics and biogeography of this group of mushrooms. Most phylogenetic studies of *Boletus* so far have characterized samples from North America and Europe and little information is available on samples from other areas, including the ecologically and geographically diverse regions of China. Here we analyzed DNA sequence variation in three gene markers from samples of these mushrooms from across China and compared our findings with those from other representative regions. Our results revealed fifteen novel phylogenetic species (about one-third of the known species) and a newly identified lineage represented by *Boletus* sp. HKAS71346 from tropical Asia. The phylogenetic analyses support eastern Asia as the center of diversity for the porcini sensu stricto clade. Within this clade, *B. edulis* is the only known holarctic species. The majority of the other phylogenetic species are geographically restricted in their distributions. Furthermore, molecular dating and geological evidence suggest that this group of mushrooms originated during the Eocene in eastern Asia, followed by dispersal to and subsequent speciation in other parts of Asia, Europe, and the Americas from the middle Miocene through the early Pliocene. In contrast to the ancient dispersal of porcini in the strict sense in the Northern Hemisphere, the occurrence of *B. reticulatus* and *B. edulis* sensu lato in the Southern Hemisphere was probably due to recent human-mediated introductions.

## Introduction

Porcini are widely collected and consumed in their main production areas in North America [1], Europe [2] and eastern Asia. The Asian populations are primarily located in China. For example, Yunnan Province in southwestern China is one of the most important centers in the world for producing, consuming and trading porcini mushrooms. Based on data from the Bolete Association of Yunnan Province, 10,572 tons of fresh boletes (porcini and other mushrooms in the order Boletales) were exported from Yunnan to other regions, primarily to Europe in 2010, resulting in gross sales of US$ 71.83 million, exceeding the US$ 47.35 million brought in by exporting the famous *Tricholoma matsutake* mushrooms (www.china.com.cn, February 15^th^, 2011). Although economically and ecologically very important (see below), the Chinese porcini are among the least understood biologically in comparison to those from Europe, Central and North America.

The porcini refer to the commonly collected *Boletus edulis* and its allies. These pored mushrooms have soft fleshy fruiting bodies, white flesh without color change when bruised or exposed to air, olive or olive-brown spores, and a stipe with an enlarged base and more or less reticulate surface [3]. They form ectomycorrhizal symbiotic relationships with both coniferous and deciduous trees and play important roles in the health of the trees and forest ecosystems. Among the closely related species in this group, however, the taxonomy and classification have been problematic and widely debated. Indeed, the Boletales, a basidiomycete order to which porcini belongs, has been described as among the most challenging groups by the 19th century mycologist E. M. Fries.

Although porcini are important in many aspects, studies on the taxonomy of this group have been, for the most part, regional. A recent study [4] proposed that the porcini sensu lato (s.l.) contains four lineages: porcini sensu stricto (s.s.), *“Inferiboletus”*, *“Alloboletus”* and *“Obtextiporus”*. In the porcini s.s. lineage, 19 phylogenetic species from Europe, North America, Africa, and eastern Asia were identified using molecular and/or morphological data [4], [5]. However, samples from eastern Asia have been very limited in previous studies and the global biogeographic pattern of the porcini remains little understood.

In China, 24 species have been recorded in the porcini [6]. They were identified mainly based on morphological features and predominantly used names that described samples from Europe and North America. Due to morphological plasticity both within and between species, however, morphology-based classifications of porcini are highly problematic and often show notable incongruence with molecular phylogenetic data. For example, porcini exported from Yunnan and adjacent areas to Europe are usually regarded as *B. edulis* by both fungal taxonomists and tradesmen. Phylogenetic analyses and sequence comparisons, however, have revealed that the Chinese porcini are highly divergent from the European and North American *B. edulis*. Instead, they seemed to cluster more closely with *B. reticulatus* [4], [7], [8], [9], [10], [11]. Prior to the work reported here, molecular analysis of Chinese porcini has been limited to species identification.

Compared to the long history of biogeographic studies of plants and animals, which can date back to before the time of Charles Darwin who used extensive biogeographic data in establishing his theory of evolution by natural selection, biogeographic studies of fungi have a relatively short history. The majority of these studies have relied on molecular markers. So far, most such studies have focused on taxa with broad geographic distributions, such as Hysterangiales [12], Inocybaceae [13] and Serpulaceae [14]. In contrast, the porcini mushrooms are mainly restricted to the holarctic region, making it a perfect lineage for investigating the impact of disjunction between Palearctic and Nearctic regions on fungal biogeography.

In our mushroom collecting efforts over the last ten years, many samples of the porcini were obtained in the field or bought from mushroom markets. Our field observations indicated tremendous morphological and ecological diversities in this group of mushrooms. We were unsure, however, if the morphological and ecological diversities simply result from phenotypic plasticity within a few species or genotypes. Alternatively, the variations could reflect true, yet undescribed, phylogenetic diversity. With the additional molecular sequence data accumulated over the last few years on porcini, it is now possible to compare the sequences between samples from China and those from other parts of the world so as to elucidate the possible historical distribution patterns of this group of mushrooms. In this paper, we aimed to address two specific objectives. In the first, through sequence analyses, we investigated the phylogenetic diversity of porcini mushrooms from China. Second, by comparing sequences from our study with sequences from the GenBank database, we presented evidence about the potential center(s) of origin and biogeographic patterns in this important group of mushrooms.

## Materials and Methods

### Taxa sampling

A broad survey for porcini mushrooms over China has been conducted during the last ten years and most mushrooms analyzed here were collected from southwestern, central, southeastern and northeastern China, covering the currently known distribution range of porcini in China. Additional samples were obtained from Russia, Japan and Germany for comparison purposes. In addition, a fruiting body growing in a forest of *Shorea robusta* in Bangladesh was included in our analyses. A brief summary about sampling sites is shown in Fig. S1 and a plate containing representatives of the species used in this study is given in Fig. S2. No specific permits were required for the described field studies, as no endangered or protected species were sampled, and the localities where the samples came from are not protected in any way. Voucher specimens for these samples are kept in the Cryptogamic Herbarium (HKAS) of the Kunming Institute of Botany, Chinese Academy of Sciences, and in the Herbarium of Mycology, Jilin Agricultural University (HMJAU). Fifty seven specimens were obtained and analyzed in this study, information about which is detailed in Table S1.

### DNA isolation, PCR and DNA sequencing

Total DNA was extracted from fruiting bodies dried in silica gel or from herbarium specimens using the CTAB method [15]. A total of three DNA fragments were analyzed in this study, including gene fragment coding for the largest subunit of RNA polymerase II (RPB1) and two non-protein coding regions within the nuclear ribosomal RNA gene cluster: the internal transcribed spacer (ITS) and the large nuclear ribosomal RNA subunit (nrLSU). Primer pairs ITS1/ITS4 [16] and LROR/LR7 [17] were used to amplify ITS and nrLSU, respectively. For RPB1, primer pair bol-RPB1-Afor and bol-RPB1-Crev [4] was used at the beginning. However, we failed to obtain PCR products for many samples. To improve the success rate, we designed a new primer pair based on our newly obtained sequences using the online program Primer3 [18]. These primers correspond to positions 195–214 (Bol-RPB1-1; 5′- CTCCGTAATCCACCGGAGAA-3′) in the intron between exon regions “A” and “B” and positions 1158–1177 (Bol-RPB1-2; 5′- TCTTCACTCCTCATTGCACC-3′) in the “C” exon region according to the annotated sequence of *Tylopilus ballouii* in GenBank (EU434340). PCR reactions were conducted on an ABI 2720 Thermal Cycler (Applied Biosystems, Foster City, CA, USA) or an Eppendorf Master Cycler (Eppendorf, Netheler-Hinz, Hamburg, Germany) and the reaction conditions were as follows: pre-denaturation at 94°C for 3 min, then followed by 35 cycles of denaturation at 94°C for 40 s, annealing at 48°C (for ITS), 50°C (for nrLSU) or 53°C (for RPB1) for 40 s, elongation at 72°C for 90 s, a final elongation at 72°C for 8 min was included after the cycles. PCR products were purified with a Gel Extraction & PCR Purification Combo Kit (Spin-column) (Bioteke, Beijing, China). The purified products were then sequenced on an ABI-3730-XL DNA Analyzer (Applied Biosystems, Foster City, CA, USA) using the same primers as in the original PCR amplifications.

### Sequence alignments

Two datasets, RPB1-nrLSU and ITS, were compiled for different analytical purposes. RPB1 and nrLSU sequences, which are more conserved than ITS, were combined into a single dataset for the phylogeny reconstruction on the porcini s.l. Meanwhile, as more porcini ITS sequences are readily available in GenBank than sequences for the other two above-mentioned genes, the diversity of species in the porcini s.s. and their relationships among each other were further investigated using the ITS dataset.

To assemble the combined RPB1-nrLSU dataset, RPB1 and nrLSU sequences used in reference [4] were retrieved and combined with our own data. Sequences for each gene were aligned separately in PRANK [19] using default parameters and manually optimized on BioEdit v7.0.9 [20]. The resulting two alignments (one for RPB1, one for nrLSU) were then concatenated using Phyutility [21] for further phylogenetic analysis. Some ambiguously aligned regions were detected in the nrLSU partition despite the optimization criteria used. These regions were characterized by the presence of gaps and uncertain positions, and were therefore excluded from subsequent analyses.

The ITS dataset consisted of our own data generated in this study and the related ITS sequences from GenBank. We first surveyed GenBank for *Boletus* using the genus search tool complement in emerencia (http://www.emerencia.org/). Both fully and insufficiently identified sequences were retrieved [22], [23] and then combined with our own sequences. Taxa outside of the porcini s.s., or with little information due to short sequence lengths (including one sequence generated from a sample named “*Boletus variipes*” collected in Thailand, which represented one phylogenetic species in reference [4]), were discarded after preliminary alignment using the –auto option in Mafft v6.8 [24]. The remaining sequences were then re-aligned using the E-INS-i option and manually edited on 4SALE v1.5 [25]. The realigned matrix contained a total of 275 sequences, about 60% of which showed minimal divergences from *B. edulis*, *B. pinophilus*, *B. aereus* and *B. reticulatus*. While not all of these sequences were included in the final phylogenetic analysis, the information about these sequences including their collection sites was used in our discussion.

### Phylogenetic analyses

Maximum likelihood (ML) analysis and Bayesian inference (BI) methods were used to analyze the two compiled datasets. Substitution models suitable for each partition in both datasets were determined using Akaike Information Criterion (AIC) implemented in MrModeltest v2.3 [26]. RAxML v7.2.6 [27] and MrBayes v3.1.2 [28] were used for ML and BI analyses respectively. The partitioned mixed model, which allows for model parameters estimated separately for each gene, was used in both the RPB1-nrLSU and the ITS analyses. The ITS alignment was divided into three partitions, ITS1, 5.8S and ITS2. The selected substitution models for these five partitions are as following: HKY+G+I for RPB1, GTR+G+I for LSU, K80+G for 5.8S, and HKY+G for both ITS1 and ITS2. All parameters in the ML analysis used the default setting, and statistical support values were obtained using nonparametric bootstrapping with 1000 replicates. BI analyses using selected models and 4 chains were conducted by setting generations to 5 million and using the stoprul command with the value of stopval set to 0.01. Trees were sampled every 100 generations. Chain convergence was determined using Tracer v1.5 (http://tree.bio.ed.ac.uk/software/tracer/) to ensure sufficiently large ESS values (>200). Burn-ins were then determined by checking the −lnL trace plots in Tracer. Subsequently, sampled trees were summarized with burn-ins discarded by using the “sumt” command implemented in MrBayes.

### Phylogenetic species determination

Because many samples from Europe and North America lacked the RPB1 and nrLSU sequences in the databases, the identification of species within the porcini s.s. lineage was based on the ITS dataset (which contained many more taxa), using criteria established to define phylogenetic or environmental species in previous studies [29], [30], [31]. In these studies, ITS sequence variations among strains within each of several known species were compared and a cutoff value was then proposed to define species limits. Specifically, in our study, based on the phylogenetic tree generated from the ITS data, each terminal branch with a high statistical support was treated provisionally as one species, same as that used in reference [4]. Intra- and inter-specific variations of these provisionally adopted species were then calculated in MEGA 5 [32] using the Maximum Composite Likelihood model [33]. Due to obvious sequence length variations among the Aereus-, Edulis- and Variipes-clades inferred in this study, we extracted the clade-specific alignment from the aligned ITS matrix for each major clade and eliminated the gap-only sites. Intra- and inter-specific variations in species within individual major clades were calculated using these alignments separately. Subsequently, five species, *B. edulis*, *B. pinophilus*, *B. aereus*, *B. reticulatus* and *B. reticuloceps*, were used to identify the range of intra-specific variation and establish a conservative cutoff value for phylogenetic species identification, with the highest intraspecific variation of these five species chosen as our cutoff value. These species were selected here because they have been well studied both morphologically and phylogenetically by other researchers [5] or by our research group (unpublished data). Any provisionally adopted species was accepted as a valid species if it showed greater divergence from its closest sister taxa than the cutoff value. In contrast, the taxa with divergence lower than the cutoff value were considered as belonging to the same species.

### Divergence time estimation

Due to the limited and sporadic fossil records in fungi, it has been difficult to choose a reliable calibration point for the divergence time estimations of any fungal group. Previous molecular study on the porcini [4] used the divergence between Basidiomycota and Ascomycota as a calibration (either 452 Mya [34] or 582 Mya [35]), derived by different researchers using different methods but based on the same 400 Million year old fossil, *Paleopyrenomycites devonicus* [36]. The different estimates were due to placing of this fossil in different subphyla in Ascomycota. However, the 452 Mya value was recently revised by placing *P. devonicus* in subphylum Pezizomycotina, and the revised value is close to 582 Mya [37]. Therefore, it seems inappropriate to use the 452 Mya value as the divergence time between Basidiomycota and Ascomycota. In this study, we used the following three calibrations: (i) the divergence between Ascomycota and Basidiomycota, (ii) the initial diversification of the mushroom forming fungi (based on the 90 million year old fossil, *Archaeomarasmius leggetti* [38]); and (iii) the divergence between Hymenochaetaceae and Fomitopsidaceae (based on the 125 million year old fossil, *Quatsinoporites cranhamii* [39]). For the first calibration, a normal distribution was applied by setting the mean and the standard deviation to 582.5 and 50.15, respectively. For the other two calibrations, the following lognormal settings were used: (i) logmean = 2.5, logstdev = 0.5, and offset = 90.0 and (ii) logmean = 2.0, logstdev = 0.5, and offset = 125.0 [14]. As the identifications of those fossils were largely ambiguous, the performances of analyses based on these calibrations were constrained by two values: (i) the minimal age of divergence between Ascomycota and Basidiomycota is at least 400 Mya, the age of *P. devonicus*; and (ii) the initial diversification of Boletales should be close to 189 Mya, the conservative estimated divergence time for the plant family Pinaceae [40], as co-estimation of divergence time for fungi and plants has indicated that the divergence between Boletales and its allies occurred slightly later than or at the similar time as the diversification of Pinaceae [41]. The calibration point that produced results to meet these criteria the closest was eventually chosen for our analyses.

To construct RPB1 and nrLSU datasets for these analyses, all RPB1 and nrLSU sequences used in reference [4] were retrieved except for those of *Glomus mosseae* and *Paraglomus occultum*, as the RPB1 sequences from these two species had part of the gene region different from those in other species. Furthermore, we retrieved sequences of four additional species, *Marasmius rotula, Mycena plumbea, M. aurantiidisca* and *Fomitopsis pinicola*, as representative taxa for the second and third calibration points. To make a robust alignment for the RPB1 dataset, introns were firstly deleted and eventually excluded from analyses. Sequences containing only exons were aligned using PRANK and manually edited when necessary. The procedure used to align nrLSU was the same as those used in our phylogenetic analyses. The BEAST 1.6.1 [42] software package was used to estimate divergence time. We first generated xml files executable in BEAST using BEAUti. RPB1 and nrLSU datasets were set as two partitions, with substitution and molecular clock models unlinked while trees were linked. For both partitions, the GTR+G model was chosen as the best substitution model by MrModelTest, and a relaxed lognormal model [43] was employed for molecular clock analysis. Tree prior was set to Yule speciation. The xml files were then executed in BEAST. For each analysis, two independent runs were conducted for 50 million generations. Log files of the two runs were combined using LogCombiner by setting the 10% logs as burn-ins and then analyzed in Tracer 1.5. The resulting trees were also combined and then interpreted in TreeAnnotator and viewed in FigTree 1.3.1 (http://tree.bio.ed.ac.uk/software/figtree/).

To further understand the diversification history of the porcini s.s., we also estimated the divergence time of the main nodes in this group using a mini ITS dataset containing representatives of all 27 species within this lineage. The porcini s.s. was inferred to have a mean crown age of 38.23 Mya (21.03–56.30 Mya, 95% HPD) by the RPB1-nrLSU data, and this value was used as a calibration point for estimating the ages of major clades by setting the prior to a normal distribution, with the mean and standard deviation set to 38.23 and 10.5, respectively, which covers the 95% HPD of the crown age. To define the most recent common ancestors (MRCAs), only the major clades supported by phylogenetic analyses based on both the RPB1-nrLSU and ITS datasets, that is, the Aereus- and Variipes-clades, were constrained as monophyletic groups. Other procedures were the same as those employed in the estimation using the RPB1-nrLSU dataset.

### Inferring the center of origin for the porcini s.s. lineage

The phylogenetic tree and estimated divergence times of the porcini s.s. lineage generated by BEAST using the mini ITS dataset were employed to reconstruct the possible historical distribution for this group. Both the Maximum Likelihood-based estimation implemented in LAGRANGE [44] and Bayesian Binary MCMC Analysis provided in RASP v2.0b [45] were used in the reconstruction efforts. The geographic distributions for the porcini s.s. were delineated into six areas: eastern Asia, eastern North America, western North America, Central American, Europe and Africa. Dispersal probabilities between areas are quite important for the reconstruction while using LAGRANGE. Corresponding to the divergence times, we employed probabilities of dispersal between different areas as summarized by reference [46] to evaluate biogeographic events of the porcini s.s. lineage. Bayesian Binary MCMC Analysis was conducted in RASP by setting generations to 10 million and with the first 10% samples discarded as burn-ins, while other parameters were kept at the default setting.

## Results

### Phylogenetics

**Figure 1:**
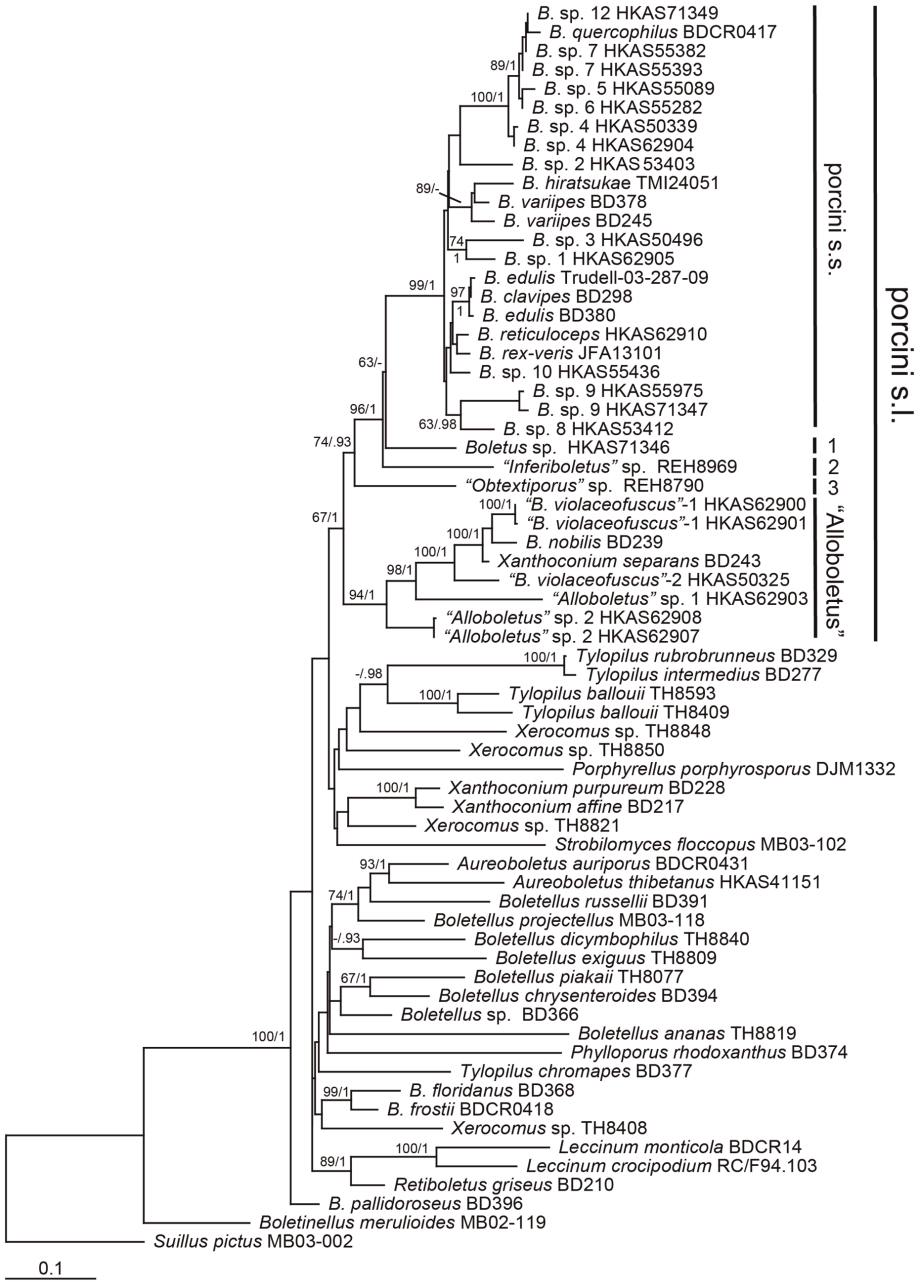
Phylogenetic tree inferred from the Maximum Likelihood (ML) analysis based on the RPB1-nrLSU data. Bootstrap values (ML)/posterior possibilities (from Bayesian Inference) are shown above or beneath individual branches. The positions of five lineages are marked with bold lines, where 1, 2, 3 represent our newly identified lineage (*Boletus* sp. HKAS71346), “*Inferiboletus*”, and “*Obtextiporus*”, respectively. All names in the porcini s.s. lineage were the same as those from ITS analysis (see Fig. 2).

The RPB1-nrLSU sequence matrix contained 66 taxa (23 were newly generated in this study) and 1348 aligned bases, with 390 bp from RPB1 and 958 bp from nrLSU. Of the 958 aligned sites in the nrLSU partition, 125 were ambiguous and were excluded from further analysis. For the RPB1 partition, an intron about 70 bp was excluded. ML and BI analyses yielded identical tree topologies and only the tree inferred from the ML analysis is shown (Fig. 1). Our result supported the porcini s.l. as a monophyletic group with a moderate bootstrap value and a high posterior probability. Within the porcini s.l. group, five monophyletic lineages, “*Alloboletus*”, “*Obtextiporus*”, “*Inferiboletus*”, *Boletus* sp. HKAS71346 and the porcini s.s., were apparent.

**Figure 2:**
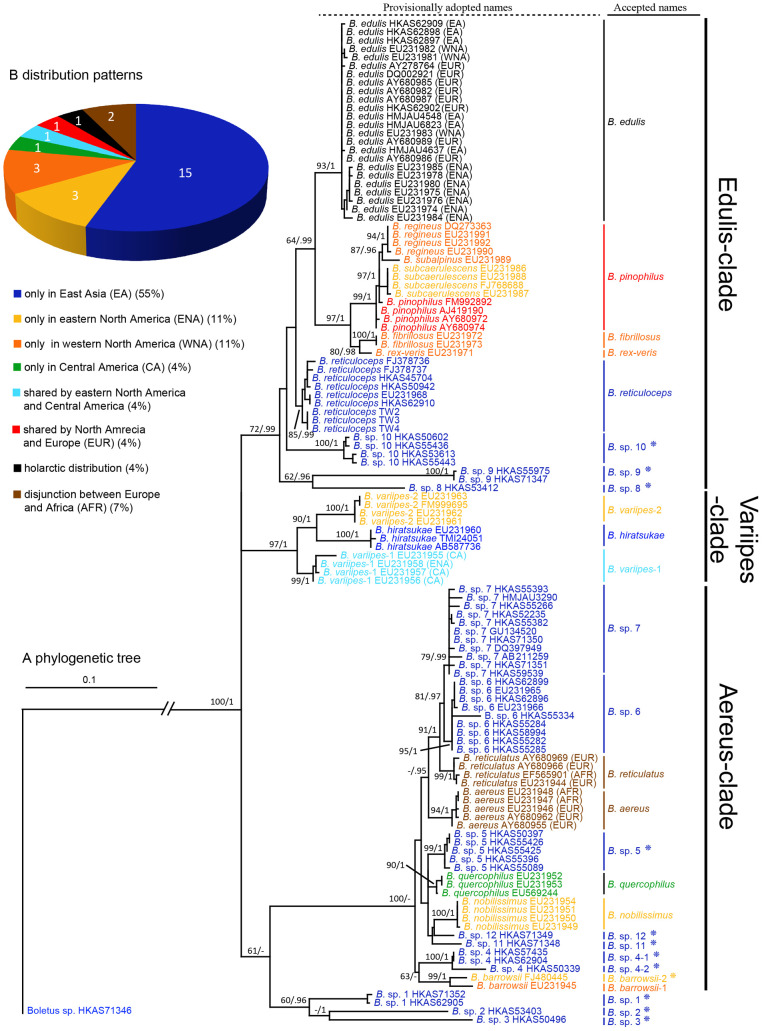
Phylogenetic tree and distribution patterns of the species within the porcini s.s. lineage. Panel A shows the topology inferred from ML analysis using the ITS data. The two values at each node represent the percentage of bootstrap supports (ML)/posterior probabilities (BI). Provisionally adopted names (based on tree topology) and finally accepted names (based on our proposed criterion for phylogenetic species identification) for all taxa are listed. Newly identified phylogenetic species are highlighted by adding asterisks after their names. Three major clades are separately marked with bold lines, and the fourth one is not marked due to its low statistical support values. Panel B lists the distribution patterns of the porcini s.s. as a pie chart. Colors in both panels represent different distribution ranges.

The final ITS sequence matrix contained 120 samples (52 were newly generated in this study) and 1236 aligned base positions. Of the 1236 positions, the ITS1, 5.8S and ITS2 partitions had 353, 159 and 724 bases respectively. The ML and BI analyses generated similar tree topologies but with some differences in bootstrap and posterior probability values. The phylogenetic tree obtained from ML analysis is shown in Fig. 2A and the tree obtained from BI is given in Fig. S3. Three major clades, named here the “Aereus-clade”, the “Edulis-clade”, and the “Variipes-clade”, could be inferred. Another clade contained three species (*B.* spp. 1–3) which have a limited distribution in China. This clade is a sister clade of the Aereus-clade, but both bootstrap support and posterior probability value were low. The positions of these three species were also uncertain in the analysis based on the RPB1-nrLSU data (Fig. 1). They showed significant divergence, however, from each other and from other taxa in the porcini s.s. lineage. Because of these observations, we prefer not to include them in any of the three major clades identified here.

### Phylogenetic species identification

As shown in Table 1, of the five species chosen as references for identifying the range of intra-specific variation of ITS sequences, *B. reticuloceps* showed the highest value (0.7%), while the lowest (0.1%) was observed in *B. pinophilus* and *B. aereus*. Thus, 0.7% was chose as the cutoff value for the phylogenetic species identification using ITS sequences. Taking this value as a threshold, *B. pinophilus*, *B. subalpinus*, *B. subcaerulescens* and *B. regineus* would be combined as one species. In contrast, our provisionally adopted *B.* sp. 4 and *B. barrowsii* showed intraspecific divergences much higher than 0.7%. These two temporarily accepted species each consisted of two sub-clades (marked with −1 and −2 respectively) in the phylogenetic tree (Fig. 2), and therefore each may represent two separate species. Other provisionally adopted species showed relatively high interspecific and low intraspecific variations and thus they were most likely valid phylogenetic species. Using this criterion, a total of 27 phylogenetic species (Fig. 2) were identified in the porcini s.s. lineage.

**Table 1:** Average evolutionary divergence over ITS sequence pairs within and between groups (provincially adopted phylogenetic species) calculated by MEGA 5 using the Maximum Composite Likelihood model.

Provincially adopted species	Interspecific variations (between groups)										Intraspecific Variations (Within groups)
*B.* sp. 9											0.002
*B.* sp. 8	0.092										n/c
*B. reticuloceps*	0.081	0.064									0.007
*B.* sp. 10	0.092	0.074	0.029								0.005
*B. edulis*	0.079	0.074	0.029	0.044							0.002
*B. rex-veris*	0.082	0.078	0.049	0.055	0.040						n/c
*B. fibrillosus*	0.082	0.073	0.045	0.050	0.040	0.009					0
*B. pinophilus*	0.086	0.074	0.045	0.054	0.032	0.019	0.019				0.001
*B. subalpinus*	0.088	0.076	0.047	0.057	0.033	0.021	0.021	0.003			n/c
*B. regineus*	0.088	0.071	0.047	0.057	0.033	0.020	0.020	0.003	0.005		0
*B. subcaerulescens*	0.086	0.074	0.050	0.060	0.036	0.023	0.023	0.006	0.007	0.007	0.001
*B. nolilissimus*											0.001
*B. quercophilus*	0.022										0.001
*B.* sp. 4	0.042	0.029									0.018
*B.* sp. 12	0.017	0.016	0.028								n/c
*B.* sp. 5	0.024	0.012	0.032	0.019							0.003
*B. barrowsii*	0.037	0.032	0.042	0.027	0.031						0.015
*B. aereus*	0.027	0.014	0.030	0.020	0.016	0.032					0.001
*B. reticulatus*	0.028	0.021	0.036	0.024	0.020	0.036	0.015				0.003
*B.* sp. 11	0.029	0.024	0.043	0.022	0.026	0.039	0.025	0.028			n/c
*B.* sp. 7	0.029	0.021	0.037	0.023	0.020	0.036	0.018	0.014	0.031		0.007
*B.* sp. 6	0.023	0.017	0.037	0.020	0.020	0.036	0.018	0.013	0.027	0.010	0.002
*B. variipes*-1											0.007
*B. variipes*-2	0.039										0
*B. hiratsukae*	0.051	0.046									0.003

### Divergence time estimation

Analyses calibrated by *Archaeomarasmius leggetti* and *Quatsinoporites cranhamii* fossils estimated the divergence time between Ascomycota and Basidiomycota as 392.48±2.94 Mya (249.69–559.54 Mya, 95% HPD) and 324.21±1.76 Mya (233.38–421.88 Mya, 95% HPD), respectively. Although their 95% HPD covered the minimal age for the Ascomycota/Basidiomycota divergence, their mean values were both less than 400 Mya. Meanwhile, these two analyses estimated the crown age of Boletales to 106.33±0.52 Mya (75.42–143.12 Mya, 95% HPD) and 86.61±0.39 Mya (65.28–108.09 Mya, 95% HPD), respectively. All of these values were significantly lower than 189 Mya. Thus, these two calibration points could vastly underestimate the divergence time of the porcini. The 582 Mya divergence calibration point between Ascomycota and Basidiomycota, however, estimated the diversification of Boletales at about 147±0.72 Mya (96.02–199.67 Mya, 95% HPD), which overlapped the conservative age for the initial diversification of Pinaceae. As a result, this calibration point was eventually applied.

**Figure 3:**
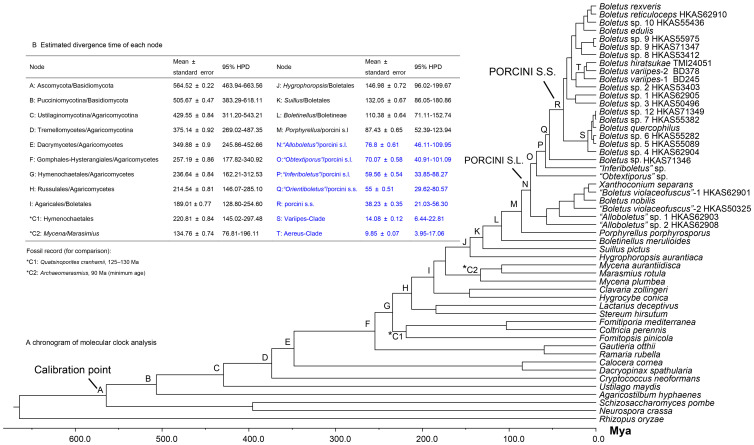
Chronogram and estimated divergence times of porcini s.l. generated from molecular clock analysis using the RPB1-nrLSU data. Chronogram obtained using the Ascomycota – Basidiomycota divergence time of 582 Mya as the calibration point is shown in panel A. The calibration point and objects of this study are marked in the chronogram. The geological time scale is in millions of years ago (Mya). Estimated divergence times of main nodes are summarized in panel B, with divergence times of lineages in the porcini s.l. highlighted in blue color.

**Figure 4:**
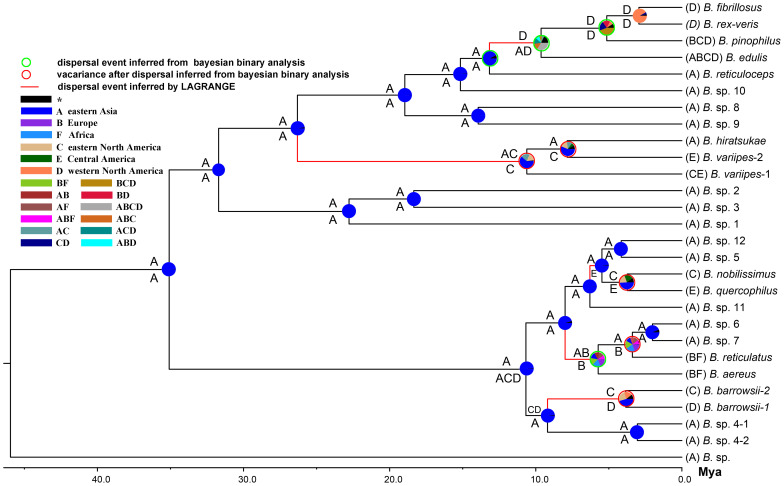
Divergence time estimation and ancestral area reconstruction of porcini s.s. using the ITS data. The Chronogram is obtained from molecular clock analysis using BEAST. Pie chart in each node indicates the possible ancestral distributions inferred from Baysian Binary MCMC analysis (BBM) implemented in RASP, while characters above and beneath each clade identify the possible ancestral distribution estimated by maximum likelihood-based program LAGRANGE. Red circles around pie charts indicate possible dispersal and vicariance events as suggested by BBM analysis, while green circles show only dispersal events. Red branches identify dispersal events inferred by LAGRANGE. Characters beyond species names show current distribution area of each species.

The chronogram from the analysis using the 582 Mya calibration point for the divergence between Ascomycota and Basidiomycota is provided in Fig. 3A. The estimated divergence times among the lineages in porcini s.l. are summarized in Fig. 3B. The data indicated an ancient divergence of the porcini s.l. from allied taxa during the late Cretaceous (76.8±0.61 Mya; 46.11–109.95 Mya, 95% HPD) and with diversification of the porcini s.s. during the middle to late Eocene (38.23±0.35 Mya; 21.03–56.30 Mya, 95% HPD). The divergence of major clades within the porcini s.s. occurred mainly during the Miocene (Fig. 3, Fig. 4).

### Reconstructing the center(s) of origin

As shown in Fig. 4, both the Maximum Likelihood-based estimation and Bayesian Binary MCMC analyses strongly supported eastern Asia as the center of origin for the porcini s.s. lineage: estimated ancestral distributions for root nodes of the porcini s.s. and its major clades were restricted to eastern Asia. Taking the estimated divergence times into account, at least 4 dispersal events from eastern Asia in the Palearctic to the Nearctic (North and Central America) via the Bering Land Bridge were suggested, followed by geographic speciation within individual areas (as shown by red branches and red cycles in Fig. 4). Within the Palearctic, there were at least 2 dispersal events from eastern Asia to Europe.

## Discussion

In this study, we investigated the phylogenetic and biogeographic patterns of the porcini mushrooms. Our data identified many novel taxa from the previously understudied areas of eastern and southern Asia. We compared the Asian samples with those previously identified species from Europe, North and Central America, and North Africa. The analyses of such a comprehensive collection from the major known distribution areas of the porcini allowed an unprecedented inference of the species diversity, phylogeny, distribution pattern and the evolutionary history of this important fungal group.

### Abundant diversity of the porcini s.l. and its nomenclature

Prior to this study, the porcini s.l. were identified as a monophyletic group consisting of four lineages: (i) the porcini s.s., (ii) the *“Inferiboletus”*, (iii) the *“Obtextiporus”*, and (iv) the *“Alloboletus”* [4]. In total 19, 1, 1 and 2 phylogenetic species were reported within those four lineages, respectively. Our study significantly expanded both the number of lineages and the number of species within individual lineages. First, we identified a new monotypic lineage (marked as *Boletus* sp. HKAS71346) in the porcini s.l. (Fig. 1), which formed sister relationship with the porcini s.s. lineage. Second, the number of species within the *“Alloboletus”* lineage increased from 2 to 6. Third, within the porcini s.s. lineage, even if we merged three species from North America (*B. subcaerulscens, B. regineus* and *B. subalpinus*) to *B. pinophilus* (Fig. 2) due to their limited ITS sequence variations (Table 1), the total number of phylogenetic species in this lineage still increased significantly as 12 new phylogenetic species (Fig. 2, *Boletus* spp.1–5, 8–12 and *B. barrowsii*-2, where *B.* sp. 4 represents two species as marked with −1 and −2) were identified as belonging to this group. We should note that our estimation for the number of phylogenetic species in the porcini s.s. is likely conservative. The three North American taxa, which were combined into *B. pinophilus*, show some morphological differences from each other, as well as from *B. pinophilus*. For example, *B. subalpinus* is different from other three by having a secotioid feature. Our results suggested that ITS sequences may have evolved slower than morphological characters in this species complex. This phenomenon has been reported from some ectomycorrhizal fungi that have undergone recent adaptive radiations, such as species in *Cortinarius* [47].

With the new phylogenetic species identified by present study added in, the total number of species of the porcini s.l. and porcini s.s. increased to 36 (from the original 23, a 36% increase) and 27 (from the original 19, a 30% increase) respectively.

In addition to the increased species diversity, our analyses also provided additional insights into the ecological heterogeneity of the porcini. Two sub-basal lineages of the porcini s.l., *“Inferiboletus”* and *“Obtextiporus”*, were found to have restricted distributions in tropical Thailand and Australia respectively, consistent with previous observations [4]. Our study expanded the distribution range of *“Alloboletus”* into tropical eastern and southern Asia, with two phylogenetic species basal to this lineage uncovered from Fujian, southeastern China (“*Alloboletus*” sp. 2) and Gazipur, Bangladesh (“*Alloboletus*” sp. 1). Furthermore, our data indicated that the two Nearctic species of this lineage, *B. nobilis* and *Xanthoconium separans*, were phylogenetically close to the eastern Asian *B. violaceo-fuscus*. *Boletus violaceo-fuscus* is distributed mainly in subtropical to temperate areas from China to Japan and contains two cryptic species as inferred in this study (Fig. 1). Within the porcini s.s. lineage, wide distribution ranges were detected for different species within China, from tropical to subalpine areas (Table S1).

It should be noted that our usage of porcini s.l., as well as names proposed provisionally to accommodate some lineages in this group [4], such as “*Obtextiporus*”, “*Inferiboletus*” and “*Alloboletus*”, will not change the fact that all lineages of porcini s.l. belong to genus *Boletus*. This is because they all share several important common features with the type species *B. edulis* and the type section *Boletus*. Given the fact that *Boletus* is not monophyletic [48], [49] and should be divided into several genera, the porcini s.l should be accepted as the true “*Boletus*”.

### Possible Indo-Malayan origin of the porcini s.l

A previous study [4] estimated the initial radiation of the porcini at about 34.4–44.0 Mya, and suggested a paleotropical origin of the group. Our study puts this date back to about 76.8 Mya and provides more evidence for a tropical origin:

1. The discovery of two new taxa of *“Alloboletus”* in tropical eastern and southern Asia, together with the identification of the new lineage (*Boletus* sp. HKAS71346) from tropical southeastern China, showed that four basal and sub-basal lineages of the porcini s.l. have a paleotropical distribution, mainly scattered in tropical eastern, southeastern and southern Asia. All of these lineages diverged much earlier than the porcini s.s., except for the four phylogenetic species within *“Alloboletus”* that are distributed throughout the temperate North Hemisphere. The distribution pattern suggests that these lineages might be relics from the evolution of the porcini s.l. and the temperate species of *“Alloboletus”* might have undergone a relatively recent diversification.
2. Even within the porcini s.s., a lineage that underwent recent radiation (38.23±0.35 Mya), some tropical species were identified. The basal taxa of the Edulis-clade, *B.* sp. 8 and *B.* sp. 9, were found from subtropical Hunan and tropical Hainan in China. These two species showed significant divergence from other taxa in the Edulis-clade, suggesting again that they were likely old relics.

These observations strongly support tropical eastern, southeastern and southern Asia as playing an important role in the early diversification of the porcini s.l. The findings here are similar to those observed in the evolution of the ant genus *Myrmica* [50], indicating a possible Indo-Malayan origin of this fungal group.

Special attention, however, should be paid to the occurrence of *“Inferiboletus”* in northeastern Australia. The estimated time of divergence for this lineage was at about 59.56±0.54 Mya (Fig. 3). While our estimations of divergence times may change with better fossil records, support for *“Inferiboletus”* as an old Gondwanan relic lineage is significant and unlikely to change. The ancient divergence times of the porcini s.l. and its three sub-basal lineages also indicate the possibility of a Gondwanan origin of this fungal group. These findings are similar to those observed for Hysterangiales [12], an ectomycorrhizal fungal Order. Under this scenario, the “Out of Africa” hypothesis cannot be rejected. The Gondwanan origin hypothesis was also proposed for the plant family Dipterocarpaceae, which likely plays a significant role as a host plant in the formation of ectomycorrhizae for some boletes [51].

There are several pieces of evidence supporting the “Out of Africa” hypothesis for the origin of the lineage *“Alloboletus”*, the most basal group of the porcini s.l. First, *“Alloboletus”* sp. 1, collected under trees of *Shorea robusta* from Bangladesh, is the first member of the porcini mushrooms reported to be apparently in association with Dipterocarpaceae. Second, fossils of *Dipterocarpus* have been found in Zhangzhou County, Fujian Province [52], where *“Alloboletus”* sp. 2 was found. Third, many boletes have been reported from wooded savannas and rain forests from Gabon, Cameroon, Zambia and Madagascar [53]. More extensive sampling from tropical Africa and its adjacent regions are needed in order to fully test the “Out of Africa” hypothesis for the porcini s.l. mushrooms.

### Biogeographic hypothesis of the porcini s.s.: old lineage with recent radiation

Our study estimated the maximum crown age of the porcini s.s. at about 38.23±0.35 Mya (Fig. 3) and strongly supported an Asian origin for the group (Fig. 4). The porcini s.s. showed a more recent diversification than its allied lineages. The difference in the diversification pattern in the four lineages within the porcini s.l. may be related to their adaptations to different ecological conditions. Most species of “*Obtextiporus*”, *“Inferiboletus”*, and *“Alloboletus”*, as well as the new lineage (*Boletus* sp. HKAS71346), are restricted to tropical areas, indicating that they have likely adapted to tropical environments, while species in the porcini s.s. have much wider distribution ranges from tropical to temperate and even subalpine areas. The four species in *“Alloboletus”* with disjunction between the Palearctic and Nearctic are also widely found in temperate regions. At the boundary of the Eocene and Oligocene (about 34 Mya), the rapid drop in temperature [54] likely led to an increase in many low temperature-adapted biota [50], [55]. Our estimation of the initial diversification of the porcini s.s. is consistent with the climate change pattern. Thus, adaptation of species to cool habitats could have accelerated the divergences of this lineage. After this initial change, taxa in this group dispersed and speciated in the northern temperate regions, contributing to their modern distribution patterns.

There are several notable features about the distribution patterns of the species within the porcini s.s. lineage. First, of the 27 phylogenetic species identified in this lineage, 15 (55%) and 8 (30%) species are endemic to eastern Asia and North/Central America, respectively. The remaining 15% are (i) *B. aereus* and *B. reticulatus* that have a European-North African disjunction, (ii) *B. pinophilus* with a European-North American disjunction, and (iii) *B. edulis*, the only species with a holarctic distribution (Fig. 2, Fig. 4). Second, of the 8 species endemic to North/Central America, 3, 3, and 1 species are found only in eastern North America, western North America and Central America respectively. Third, 13 of 14 species endemic to eastern Asia have been, to the best of our knowledge, reported only from China. We should note that additional samples from these and other areas might reduce the number of endemic species in this lineage. However, as shown in this study, additional new taxa and evidence for endemism may also increase. Below we discuss the potential mechanisms for the high incidences of endemism in different geographic areas.

Eastern Asia is the center of origin for the porcini s.s. lineage and holds many old relics which contribute to the high endemism of porcini mushrooms in this region. These relics typically have restricted and heterogeneous distribution ranges. Of the four basal and sub-basal species of the Edulis-clade, *B.* sp. 8 and *B.* sp. 9 are from subtropical Hunan and tropical Hainan, while *B.* sp. 10 and *B. reticuloceps* are mainly restricted to subalpine areas in southwestern China, with the latter occupying an expanded range to relatively high elevations in central China and even to subalpine regions in Taiwan. After the uplift of the Tibetan plateau due to the collision of the Indian and Eurasian plates [56], stochastic changes in temperature may have led to the expansion and contraction of populations of many organisms to small regions (refugia) in eastern Asia [55]. Central, southern and southwestern China contained important refugia for many plant species during Earth's recent geological history [57]. Thus, the diverse ecological distribution of these old relics in the Edulis-clade could have resulted from these paleoclimate changes.

The dispersal-vicariance theory has been widely used to explain disjunctions of plants between Palearctic and Nearctic regions [58]. This theory was also used to explain the biogeographic pattern of the fly agaric *Amanita muscaria* [59], [60]. Similar to these studies, our results suggested that multiple dispersals via the Bering Land Bridge and regional speciation after vicariance due to changing climate conditions during the middle Miocene through early Pliocene likely contributed significantly to the occurrences and endemism of porcini mushrooms in Central and North America. Ancestral area reconstructions using LAGRANGE and RASP both suggested that many species distributed in North/Central America have counterparts in eastern Asia, and that they likely shared the same ancestors that originated in eastern Asia. For example, in the Aereus-clade, the distribution of *B. barrowsii*-1 and -2 in western and eastern North America could be the result of one dispersal event from eastern Asia to North America. Similarly, the occurrence of *B. noblissimus* and *B. quercophilus* in eastern North America and Central America could be attributed to another dispersal event. In the Edulis- and Variipes- clades, at least two dispersal events were likely involved in the disjunction of species or species pairs between Palearctic and Nearctic regions. Although no evidence for the co-dispersal of these ectomycorrhizal fungi with their host plants has been reported, the continuous distribution of temperate coniferous forests in northwestern North America and northeastern Asia from the Miocene to the Pliocene [61], [62] could have mediated the exchange of ectomycorrhizal fungal biota between the Palearctic and the Nearctic.

### Human activity-mediated introduction of porcini mushrooms into novel habitats

Here we discuss the disjunctions of *B. aereus* and *B. reticulatus* between Europe and Africa separately, as they likely represent two different ways for biota to invade new habitats through natural dispersal or human activity-mediated introductions.

*Boletus aereus* has a continuous distribution from southern Europe to northern Morocco and these populations share the same host plants. *Quercus suber*, an important host plant for *B. aereus*, shows a similar distribution patterns as its fungal partner, and is believed to have a natural uninterrupted distribution from southern Europe to northern Africa [63]. Thus, the occurrence of *B. aereus* in Africa could be the result of natural dispersal from Europe to northern Africa along with its host plants.

Our study is the first to report *B. reticulatus* from southern Africa. Another member of the porcini s.s. found in the Southern Hemisphere is *B. edulis* s.l. from New Zealand [64]. These two taxa live with their non-native host plants, thus their occurrence in the Southern Hemisphere is likely due to their recent dispersals coupled with introduction of their host plants to these areas [31], [65], [66]. In particular, *B. reticulatus* in South Africa is associated with *Pinus patula*, a pine native to Mexico and introduced to South Africa. *Boletus reticulatus* was originally described from Europe and was found to have relatively low host plant specificity. In addition, no record of this mushroom from Mexico or adjacent areas has been reported. Introduction of pines around the world during former colonial times sometimes followed research on climate-appropriate trees in experimental agricultural stations in which multiple species were grown together. In such experimental settings or after introduction of multiple species of pine, fungi with low host specificities could readily adapt to new hosts. These factors may have contributed to the co-occurrence of European fungi and Central American plants interacting with each other in South Africa.

### Conclusions

A comprehensive phylogenetic study of the porcini was conducted using sequences of three loci with an emphasis on new samples from eastern Asia. Distribution patterns and historical biogeography were revealed and discussed. An unexpected high species count and significant ecological diversity in the porcini were found and a possible Indo-Malayan origin of the porcini s.l. was suggested. Our study also identified eastern Asia as the center of origin for the porcini s.s. lineage. Samples from tropical regions were found especially informative for the inferences of evolution and historical biogeography of the porcini. Materials from the tropics, however, are still limited. An international collaboration of mycological researchers from several continents and broader and more extensive sampling and analysis will help to resolve some of the unanswered questions in this group of economically and ecologically important mushrooms.

## Supporting Information

Table S1 Taxon information and GenBank accession numbers for the sequences used in this study.
(DOC)

Figure S1 A map showing sites for porcini sampling in eastern and southern Asia.
(TIF)

Figure S2 A plate containing representatives of undescribed porcini mushrooms used in this study.
(TIF)

Figure S3 Phylogenetic tree of the porcini s.s. generated from ITS dataset using Bayesian Inference (BI).
(TIF)
