# Correction: DNA Sequence Analyses Reveal Abundant Diversity, Endemism and Evidence for Asian Origin of the Porcini Mushrooms

**DOI:** 10.1371/annotation/e918501c-a3a9-4c14-9ea6-bcc6cf53062e

**Published:** 2012-08-13

**Authors:** Md. Iqbal Hosen, Bang Feng, Jianping Xu, Gang Wu, Nian-Kai Zeng, Yan-Chun Li, Bau Tolgor, Gerhard W. Kost, Zhu L. Yang

Md. Iqbal Hosen was not included in the author byline. He should be listed as the fourth author and affiliated with 1. Key Laboratory of Biodiversity and Biogeography, Kunming Institute of Botany, Chinese Academy of Sciences, Kunming, Yunnan, People's Republic of China, and 5. Graduate University of Chinese Academy of Sciences, Beijing, People's Republic of China. The contributions of this author are correctly shown in the Author Contributions section.

The correct citation is: Feng B, Xu J, Wu G, Hosen MI, Zeng N-K, et al. (2012) DNA Sequence Analyses Reveal Abundant Diversity, Endemism and Evidence for Asian Origin of the Porcini Mushrooms. PLoS ONE 7(5): e37567. doi:10.1371/journal.pone.0037567 

