# Supplementary material for: DNA Sequence Analyses Reveal Abundant Diversity, Endemism and Evidence for Asian Origin of the Porcini Mushrooms
Source: PLoS One. 2012 May 18;7(5):e37567. doi: 10.1371/journal.pone.0037567 (PMC3356339; doi:10.1371/journal.pone.0037567)
Supplement: Table S1 — Taxon information and GenBank accession numbers for the sequences used in this study. (DOC) [file pone.0037567.s001.doc]

Table S1 Taxon information and GenBank accession numbers for the sequences used in this study

| Taxon | Herbarium ID | Collection  ID | Location | GenBank accession numbers | | |
| --- | --- | --- | --- | --- | --- | --- |
|  |  |  |  | ITS | LSU | RPB1 |
| “*Alloboletus*” sp. 1 | HKAS62903 | Hosen14 | On ground under *Shorea robusta*, Bhawal National Park, Gazipur, Bangladesh | - | JN563855 | JN563875 |
| “*Alloboletus*” sp. 2 | HKAS62907 | Zeng639 | Under forest of *Lithocarpus* spp. and *Castanopsis* spp., Zhangping County, Fujian Province, China | - | JN563856 | JN563873 |
| “*Alloboletus*” sp. 2 | HKAS62908 | Zeng652 | Under forest of *Lithocarpus* spp. and C*astanopsis* spp., Zhangping County, Fujian Province, China | - | JN563857 | JN563874 |
| *Boletus edulis* | HKAS62897 | Shi473 | Changbai Mountain, Antu County, Jilin Province, China | JN563889 | - | - |
| *Boletus edulis* | HKAS62898 | Shi640 | On ground under *Pinus sylvestris* var. *mongolica*, Honghuaerji National Forest Park, Hulunbeier, Inner Mongolia, China | JN563890 | - | - |
| *Boletus edulis* | HKAS62902 | Yang5025 | On ground under *Pinus sylvestris* and *Betula* spp., Marburg Botanical Garden, Germany | JN563891 | - | - |
| *Boletus edulis* | HKAS62909 | Wang2692 | On ground under *Larix gmelinii* and *Betula ermanii*, Dabaishan Mountain, Huzhong County, Heilongjiang Province | JN563888 | - | - |
| *Boletus edulis* | HMJAU4548 | Tolgor Bau | Orlovsky, Kyrov, Russia | JN563892 | - | - |
| *Boletus edulis* | HMJAU4637 | Tolgor Bau | Belaya Holuntsa, Kyrov, Russia | JN563894 | - | - |
| *Boletus edulis* | HMJAU6823 | Tolgor Bau | From free market in A’ershan, Inner Mongolia, China | JN563893 | - | - |
| *Boletus hiratsukae* | TMI24051 | H. Suga | Under *Picea jezoensis* var. *hondensis*, Asahiyama Park, Sapporo, Japan | JQ172780 | JQ172788 | JQ172793 |
| *Boletus reticuloceps* | / | TW2 | On Ground under *Tsuga* spp., Hehuan Mountain, Nantu County, Taiwan, China | JN563887 | - | - |
| *Boletus reticuloceps* | / | TW3 | On Ground under *Tsuga* spp., Hehuan Mountain, Nantu County, Taiwan, China | JN563886 | - | - |
| *Boletus reticuloceps* | / | TW4 | On Ground under *Tsuga* spp., Hehuan Mountain, Nantu County, Taiwan, China | JN563885 | - | - |
| *Boletus reticuloceps* | HKAS45704 | Yang4325 | On ground under *Picea* spp., Leiwuqi County, Tibet, China | JN563883 | - | - |
| *Boletus reticuloceps* | HKAS50942 | Ge1355 | On ground under remnant *Picea* forest, on the way from Baiyu to Batang, Sichuan Province, China | JN563882 | - | - |
| *Boletus reticuloceps* | HKAS62910 | Zhangping540 | Shennongjia, Hubei Province, China | JN563884 | JN563843 | JN563862 |
| *Boletus* sp. | HKAS71346 | Zeng619 | Zhangping County, Fujian Province, China | JQ172779 | JQ172789 | JQ172792 |
| *Boletus* sp. 1 | HKAS62905 | Feng792 | Under *Lithocarpus* spp. and *Quercus* spp., Kunming, Yunnan Province, China | JN563924 | JN563844 | JN563865 |
| *Boletus* sp. 1 | HKAS71352 | Feng1183 | Qiongzhu Temple, Kunming, Yunnan Province, China | JQ172785 | - | - |
| *Boletus* sp. 2 | HKAS53403 | Li1058 | Mangshan Mountain, Yizhang County, Hunan Province, China | JN563925 | JN563845 | JN563866 |
| *Boletus* sp. 3 | HKAS50496 | Yang4699 | Ailao Mountain, Jingdong County, Yunnan Province, China | JN563923 | JN563846 | JN563864 |
| *Boletus* sp. 4 | HKAS50339 | Li585 | Ailao Mountain, Jingdong County, Yunnan Province, China | JN563922 | JN563847 | JN563871 |
| *Boletus* sp. 4 | HKAS57435 | Feng706 | On ground under *Lithocarpus* and Q*uercus*, Tianwentai, Yulong County, Yunnan Province, China | JN563921 | - | - |
| *Boletus* sp. 4 | HKAS62904 | ZQ8194 | Tianwentai, Yulong County, Yunnan Province, China | JN563920 | JN563848 | JN563872 |
| *Boletus* sp. 5 | HKAS50397 | Li643 | On ground under *Pinus* spp. and *Quercus* spp., near Hongshan Mountain, Shangeri-La County, Yunnan Province, China | JN563919 | - | - |
| *Boletus* sp. 5 | HKAS55089 | ZQ888 | Heibaishui, Yunlong County, Yunnan Province, China | JN563918 | JN563849 | JN563869 |
| *Boletus* sp. 5 | HKAS55396 | Feng285 | On ground under *Pinus densata*, Yunlong Snow Mountain, Yulong County, Yunnan Province, China | JN563916 | - | - |
| *Boletus* sp. 5 | HKAS55425 | Feng314 | On ground under *Pinus densata* and *Quercus aquifolioides*, Haba Snow Mountain, Shangeri-La County, Yunnan Province, China | JN563915 | - | - |
| *Boletus* sp. 5 | HKAS55426 | Feng315 | On ground under *Pinus densata* and *Quercus aquifolioides*, Haba Snow Mountain, Shangeri-La County, Yunnan Province, China | JN563914 | - | - |
| *Boletus* sp. 6 | HKAS55282 | ZQ8320 | On ground under *Pinus yunnanensis*, Shitou, Yulong County，Yunnan Province, China | JN563913 | JN563850 | JN563870 |
| *Boletus* sp. 6 | HKAS55284 | ZQ8322 | Yongchun, Weixi County, Yunnan Province, China | JN563912 | - | - |
| *Boletus* sp. 6 | HKAS55285 | ZQ8323 | On ground under *Pinus yunnanensis*, Yongchun, Weixi County, Yunnan Province, China | JN563911 | - | - |
| *Boletus* sp. 6 | HKAS55334 | Feng224 | On ground under *Pinus yunnanensis,* near Lashi Lake, Yulong County, Yunnan Province, China | JN563917 | - | - |
| *Boletus* sp. 6 | HKAS58994 | Zhao531 | Tuozhi, Weixi County, Yunnan Province, China. | JN563909 | - | - |
| *Boletus* sp. 6 | HKAS62896 | Shi413 | Neixiang Village, Nanyang County, Henan Province, China | JN563908 | - | - |
| *Boletus* sp. 6 | HKAS62899 | Shi676 | On ground under *Pinus yunnanensis,* Luoji Mountain, Puge County, Sichuan Province, China | JN563907 | - | - |
| *Boletus* sp. 7 | HKAS52235 | Yang4918 | From free market in Nanhua County, Yunnan Province, China | JN563905 | - | - |
| *Boletus* sp. 7 | HKAS55266 | ZQ8289 | On ground under mixed forest of *Pinus* spp. and *Quercus* spp., Yulong County, Yunnan Province, China | JN563904 | - | - |
| *Boletus* sp. 7 | HKAS55382 | Feng271 | On ground under *Pinus kesiya* var. *langbianensis*, on the way from Ning'er to Pu'er, Yunnan Province, China | JN563903 | JN563851 | JN563867 |
| *Boletus* sp. 7 | HKAS55393 | Feng282 | From free market in Nanhua County, Yunnan Province, China | JN563902 | JN563852 | JN563868 |
| *Boletus* sp. 7 | HKAS59539 | Li1792 | On ground under *Pinus yunnanensis*, Wojiaodi Village, Changning County, Yunnan Province, China | JN563901 | - | - |
| *Boletus* sp. 7 | HMJAU3290 | Tolgor Bau | Fuyuan County, Heilongjiang Province, China | JN563906 | - | - |
| *Boletus* sp. 7 | HKAS71350 | Feng953 | Gongshan County, Yunnan Province, China | JQ172782 | - | - |
| *Boletus* sp. 7 | HKAS71351 | Feng959 | Gongshan County, Yunnan Province, China | JQ172783 | - | - |
| *Boletus* sp. 8 | HKAS53412 | Li1109 | Sanping, Mangshan Mountain, Yizhang, Hunan Province, China | JN563900 | JN563926 | JN563880 |
| *Boletus* sp. 9 | HKAS55975 | Li1067 | Mangshan Mountain, Yizhang, Hunan Province, China | JN563899 | JN563853 | JN563879 |
| *Boletus* sp. 9 | HKAS71347 | Zeng820 | Limu Mountain, Qiongzhong County, Hainan Province, China | JQ172784 | JQ172790 | JQ172791 |
| *Boletus* sp. 10 | HKAS50602 | Ge1018 | On ground under mixed deciduous and coniferious forest, on the way from Shangeri-La to Haba, Yunnan Province, China | JN563898 | - | - |
| *Boletus* sp. 10 | HKAS53613 | Ge1527 | On ground under remnant forest of *Larix*, scattered *Abies*, some *Picea* and shrubs, on the way from Danba to Daofu, Sichuan Province, China | JN563897 | - | - |
| *Boletus* sp. 10 | HKAS55436 | Feng325 | On ground under *Picea likiangensis*, Baima Snow Mountain, Deqin County, Yunnan Province, China | JN563896 | JN563854 | JN563863 |
| *Boletus* sp. 10 | HKAS55443 | Feng332 | On ground under *Picea likiangensis*, Baima Snow Mountain, Deqin County, Yunnan Province, China | JN563895 | - | - |
| *Boletus* sp. 11 | HKAS71348 | Shi811 | Laoshan, Qingdao, Shandong Province, China | JQ172786 | - | - |
| *Boletus* sp. 12 | HKAS71349 | Shi839 | Huaguoshan, Lianyunguang, Jiangsu Province, China | JQ172781 | JQ172787 | JQ172794 |
| *Boletus violaceo-fuscus* | HKAS50325 | Li571 | Ailao Mountain, Jingdong County, Yunnan Province, China | - | JN563858 | JN563878 |
| *Boletus violaceo-fuscus* | HKAS62900 | Wu383 | From free market in Nanhua County, Yunnan Province, China | - | JN563859 | JN563876 |
| *Boletus violaceo-fuscus* | HKAS62901 | Wu387 | From free market in Nanhua County, Yunnan Province, China | - | JN563860 | JN563877 |
