# Supplementary figures and images for: DNA Sequence Analyses Reveal Abundant Diversity, Endemism and Evidence for Asian Origin of the Porcini Mushrooms
Source: PLoS One. 2012 May 18;7(5):e37567. doi: 10.1371/journal.pone.0037567 (PMC3356339; doi:10.1371/journal.pone.0037567)

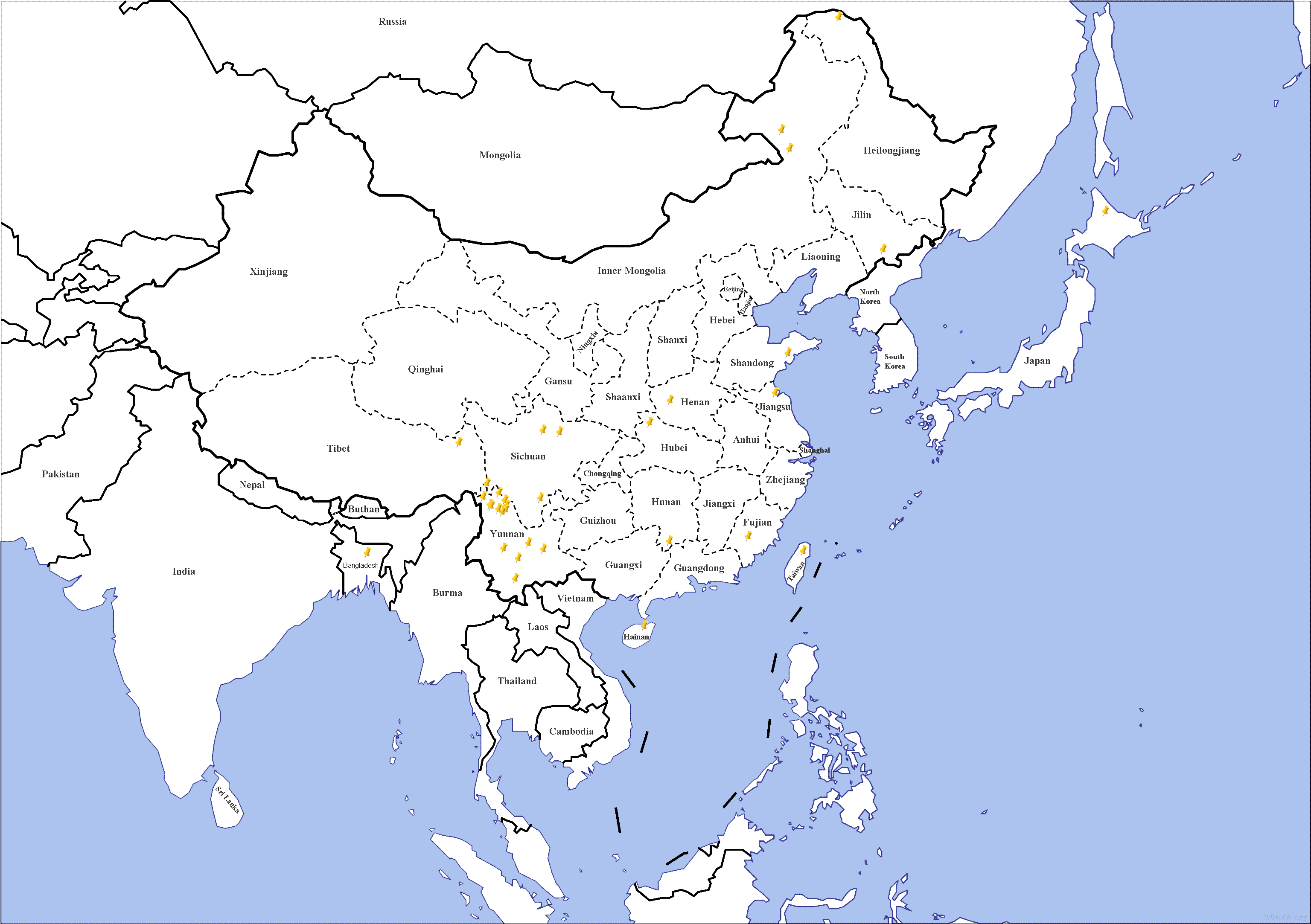

Supplement: Figure S1 — A map showing sites for porcini sampling in eastern and southern Asia. (TIF) [file pone.0037567.s002.tif]

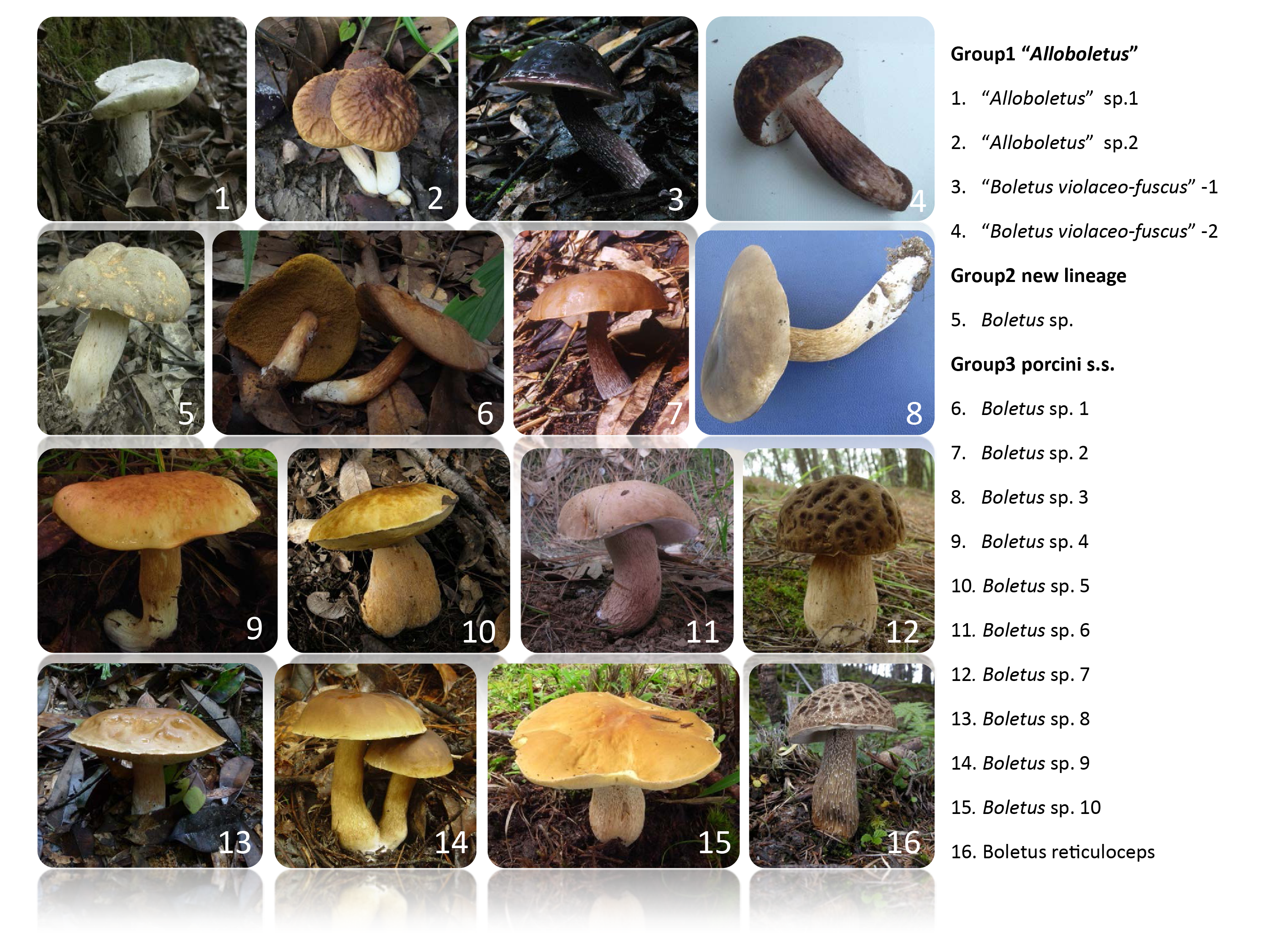

Supplement: Figure S2 — A plate containing representatives of undescribed porcini mushrooms used in this study. (TIF) [file pone.0037567.s003.tif]

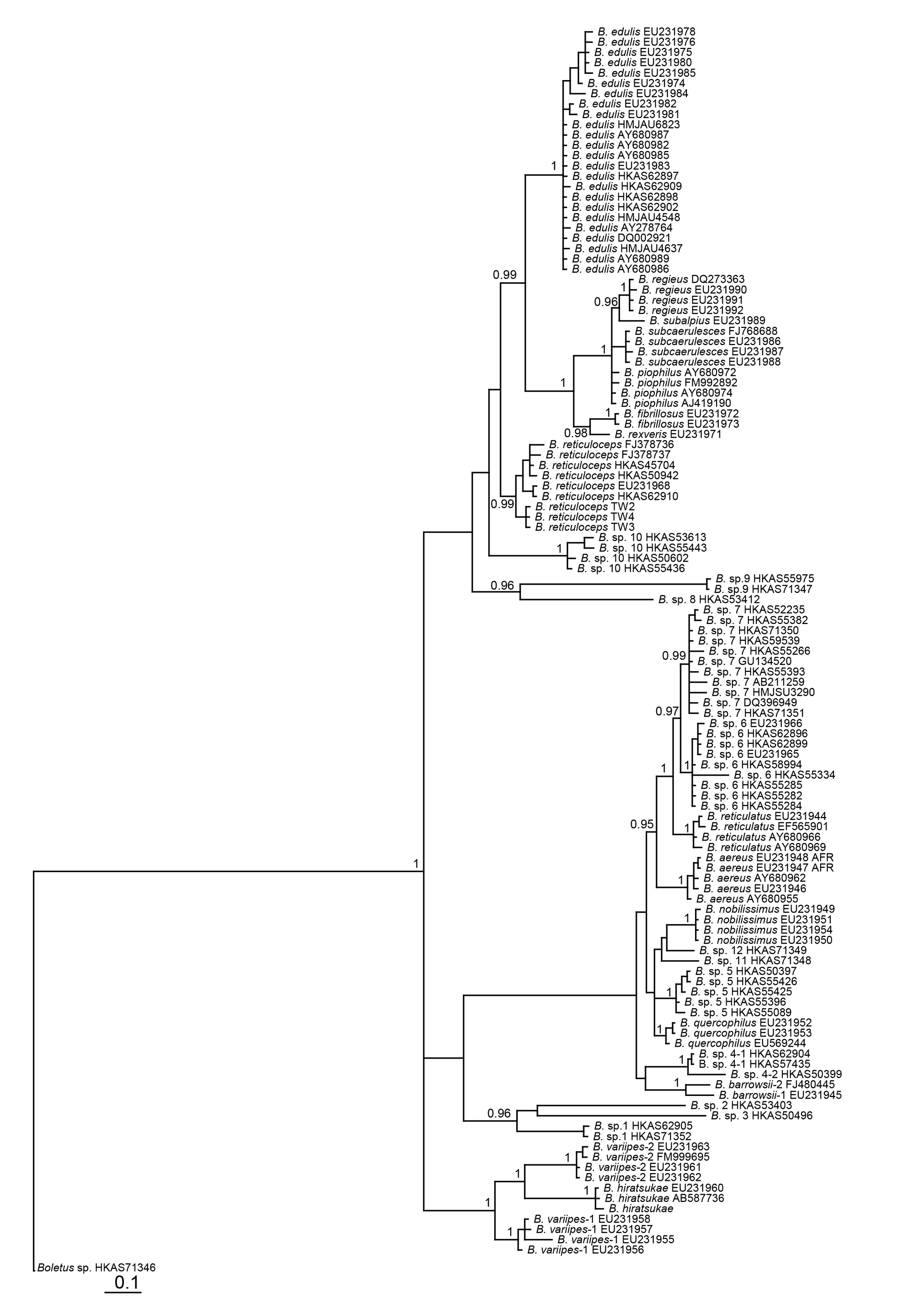

Supplement: Figure S3 — Phylogenetic tree of the porcini s.s. generated from ITS dataset using Bayesian Inference (BI). (TIF) [file pone.0037567.s004.tif]
